# Crystal structure of the middle and C-terminal domains of Hsp90α labeled with a coumarin derivative reveals a potential allosteric binding site as a drug target

**DOI:** 10.1107/S2059798322002261

**Published:** 2022-04-08

**Authors:** Shuxia Peng, Jeff Woodruff, Prabhat Kumar Pathak, Robert L. Matts, Junpeng Deng

**Affiliations:** a Department of Biochemistry and Molecular Biology, Oklahoma State University, 246 Noble Research Center, Stillwater, OK 74078, USA

**Keywords:** chaperones, Hsp90 inhibitors, crystal structure, allosteric regulation, coumarin derivatives

## Abstract

The 90 kDa heat-shock protein (Hsp90) is an abundant molecular chaperone that is essential to activate, stabilize and regulate the function of a plethora of client proteins. As drug targets for the treatment of cancer and neurodegenerative diseases, Hsp90 inhibitors that bind to the N-terminal ATP-binding site of Hsp90 have shown disappointing efficacy in clinical trials. Thus, allosteric regulation of the function of Hsp90 by compounds that interact with its middle and C-terminal (MC) domains is now being pursued as a mechanism to inhibit the ATPase activity and client protein-binding activity of Hsp90 without concomitant induction of the heat-shock response. Here, the crystal structure of the Hsp90αMC protein covalently linked to a coumarin derivative, MDCC {7-diethylamino-3-[*N*-(2-maleimidoethyl)carbamoyl]coumarin}, which is located in a hydrophobic pocket that is formed at the Hsp90αMC hexamer interface, is reported. MDCC binding leads to the hexamerization of Hsp90, and the stabilization and conformational changes of three loops that are critical for its function. A fluorescence competition assay demonstrated that other characterized coumarin and isoflavone-containing Hsp90 inhibitors compete with MDCC binding, suggesting that they could bind at a common site or that they might allosterically alter the structure of the MDCC binding site. This study provides insights into the mechanism by which the coumarin class of allosteric inhibitors potentially disrupt the function of Hsp90 by regulating its oligomerization and the burial of interaction sites involved in the ATP-dependent folding of Hsp90 clients. The hydrophobic binding pocket characterized here will provide new structural information for future drug design.

## Abstract 2

Allosteric inhibitors that bind to the middle domain (MD) or C-terminal domain (CTD) of Hsp90 have become promising drug leads for the development of effective and nontoxic chemicals in anticancer drug discovery. The structure of MDCC-labeled Hsp90α MD and CTD reported here provides the first direct visual insight into allosteric binding inhibitors of Hsp90 MD or CTD and provides a basis for the design of novel drugs for the treatment of cancer and neurodegenerative diseases.

## 1. Introduction

The 90 kDa heat-shock protein (Hsp90) is an abundant, essential cellular protein family present in all eukaryotes that is the hub of a molecular chaperone machine which utilizes a reaction cycle driven by ATP hydrolysis to stabilize, regulate and activate the function of several hundred important client proteins under both normal and stress conditions (Schopf *et al.*, 2017 ▸; Sima & Richter, 2018 ▸; Pearl, 2016 ▸). Since the overexpression or accumulation of misfolded proteins is associated with many diseases such as cancer, neurodegenerative and infectious diseases, inhibition of Hsp90 has been pursued as a promising method for their treatment (Chaudhury *et al.*, 2006 ▸; Shrestha *et al.*, 2016 ▸; Wang *et al.*, 2017 ▸; Alam *et al.*, 2017 ▸; Ekman *et al.*, 2010 ▸; Augello *et al.*, 2019 ▸; Buc Calderon *et al.*, 2019 ▸; Garg *et al.*, 2016 ▸). Hsp90 family members comprise four isoforms, including Hsp90α and Hsp90β, which are nucleo­cytoplasmic, GRP94 located in the endoplasmic reticulum (ER) and TRAP1 located in mitochondria. Hsp90β is constitutively expressed, while Hsp90α is especially induced in response to stress conditions (Biebl & Buchner, 2019 ▸; Hoter *et al.*, 2018 ▸). All of the Hsp90 homologues exist as flexible homodimers, with each protomer consisting of three domains: an N-terminal domain (NTD), a middle domain (MD) and a C-terminal domain (CTD). A dynamic highly charged linker connects the NTD and MD in cytoplasmic and ER forms of eukaryotic Hsp90 (Jahn *et al.*, 2014 ▸). The NTD contains an ATP-binding site, while the MD also participates in ATP hydrolysis by contributing a conserved arginine (Arg400 in human Hsp90α) from the ‘catalytic loop’ to stabilize the γ-phosphate of ATP (Cunningham *et al.*, 2012 ▸). The CTD is involved in homodimerization. All three domains are involved in the binding of various co-chaperones as well as client proteins (Genest *et al.*, 2019 ▸). Conformational changes of the Hsp90 dimer, with open, semi-open or closed states accompanying ATP binding and the hydrolysis and release of ADP, are modulated by the binding or release of co-chaperones, post-translational modifications and interaction with client proteins (Biebl & Buchner, 2019 ▸; Prodromou, 2016 ▸; Backe *et al.*, 2020 ▸). Hsp90 homodimers can spontaneously self-associate to form oligomers under heat-shock or even under unstressed conditions (Nemoto & Sato, 1998 ▸). A recent study showed that oligomers of Hsp90 could also participate in the Hsp90 chaperone cycle regulated by co-chaperones such as Aha1 and p23 (Lepvrier, Moullintraffort *et al.*, 2015 ▸; Lepvrier, Nigen *et al.*, 2015 ▸; Lepvrier *et al.*, 2018 ▸).

As a drug target, the most developed class of Hsp90 inhibitors, the Hsp90 NTD inhibitors (NT-inhibitors), directly bind the ATP-binding pocket to disrupt the Hsp90 chaperone cycle and lead to client-protein degradation (Khandelwal *et al.*, 2016 ▸). However, during clinical trials investigating the use of NT-inhibitors for the treatment of cancer, activation of heat-shock factor 1 (Hsf1) and induction of the pro-survival heat-shock response (HSR) were noted. The HSR-induced elevation of the levels of Hsps required dose escalation, where dose-limiting toxic side effects were noted (Trepel *et al.*, 2010 ▸; Neckers *et al.*, 2018 ▸; Hong *et al.*, 2013 ▸; Mielczarek-Lewandowska *et al.*, 2020 ▸). Isoform-selective inhibitors are now being developed to improve clinical outcomes by inhibiting the specific Hsp90 isoforms, which play overlapping but also isoform-specific roles in cells (Que *et al.*, 2018 ▸; Jiang *et al.*, 2018 ▸; Khandelwal *et al.*, 2018 ▸; Lee *et al.*, 2015 ▸; Crowley *et al.*, 2016 ▸; Mishra *et al.*, 2021 ▸).

As an alternative strategy, the MD and CTD are currently being actively investigated as potential sites for the binding of allosteric Hsp90 inhibitors. Initially, a second putative nucleotide-binding site was discovered in the CTD, which binds novobiocin and related coumarins that inhibit Hsp90 activity, and induces degradation of selective client proteins, without inducing the HSR (Donnelly & Blagg, 2008 ▸; Marcu, Chadli *et al.*, 2000 ▸; Marcu, Schulte *et al.*, 2000 ▸; Soti *et al.*, 2003 ▸; Sreedhar *et al.*, 2004 ▸). The latter property of these compounds supports the hypothesis that such drugs are likely to provide a promising strategy for the treatment of cancer (Goode *et al.*, 2017 ▸; Eskew *et al.*, 2011 ▸; Burlison *et al.*, 2008 ▸; Armstrong *et al.*, 2016 ▸). Second and third generations of CTD inhibitors with varying scaffolds have been developed that have an affinity that is higher but is not sufficient for them to be clinically viable. Hsp90 isoform-selective inhibitors that bind to the MD of Hsp90 have also been identified for anticancer drug development (Mak *et al.*, 2019 ▸; Zhang *et al.*, 2018 ▸; Yim *et al.*, 2016 ▸; Zhou *et al.*, 2020 ▸). Both the MD and CTD are now considered to contain binding sites for allosteric regulators that are distal to the ATP-binding site in the NTD, but are capable of inducing conformational changes that either activate or inhibit Hsp90 function (Rehn *et al.*, 2016 ▸; D’Annessa *et al.*, 2019 ▸; Ferraro *et al.*, 2019 ▸; Strocchia *et al.*, 2015 ▸) or disrupt the interaction of Hsp90 with its co-chaperone partners. For example, celastrol and withaferin A inhibit the interaction of Cdc37 with Hsp90 (Sreeramulu *et al.*, 2009 ▸; Zhang *et al.*, 2008 ▸; Li *et al.*, 2018 ▸; Hadden *et al.*, 2007 ▸).

Various computational approaches have been utilized to identify potential allosteric binding sites in Hsp90 (Bickel & Gohlke, 2019 ▸; Sgobba *et al.*, 2008 ▸; Khalid & Paul, 2014 ▸; Matts, Dixit *et al.*, 2011 ▸; Kumar MV *et al.*, 2018 ▸). These computationally derived structural models have predicted a number of putative allosteric binding sites in the Hsp90 MD and CTD and at the MD–CTD interface (Blacklock & Verkhivker, 2014 ▸; Penkler & Tastan Bishop, 2019 ▸; Penkler *et al.*, 2018 ▸; Vettoretti *et al.*, 2016 ▸; Sanchez-Martin *et al.*, 2020 ▸; Sgobba *et al.*, 2010 ▸), and have predicted chemical structures with which these sites might interact. However, these computational studies primarily use the closed form of Hsp90 as a template for docking, as it is the dominant conformation of full-length (FL) Hsp90 available in the PDB. Additionally, the published crystal structures lack structural information in regions of the protein that are known to be important for Hsp90 function. Thus, the development of high-affinity Hsp90 inhibitors that interact with the Hsp90 MD and CTD has been hindered by the lack of any co-crystal structures that could be exploited for rational drug design. In this study, we report the crystal structure of human Hsp90α MD and CTD (Hsp90αMC) labeled with a coumarin derivative, MDCC {7-diethylamino-3-[*N*-(2-maleimidoethyl)carbamoyl]coumarin}, which is covalently linked to Cys374 of Hsp90α. The structure identified a hydrophobic binding pocket for the coumarin scaffold, which could represent an allosteric binding site for Hsp90 inhibitors containing the common coumarin core. This structure provides the first direct visual insight into the mechanism of Hsp90 MD or CTD allosteric inhibitors, and provides a basis for the design of new drugs.

## 2. Materials and methods

### 2.1. Gene cloning, protein expression and purification

Hsp90α_293–699 (Hsp90αMC) was cloned into a modified pET vector with an N-terminal 6×His tag, including a Tobacco etch virus (TEV) protease cleavage site between the tag and the protein sequence. The recombinant protein was expressed in *Escherichia coli* and purified by Ni–NTA affinity-purification procedures as described previously (Krumm *et al.*, 2008 ▸). Briefly, Hsp90αMC was first purified from the soluble cell lysate on an Ni–NTA affinity column using loading buffer (20 m*M* Tris–HCl, 500 m*M* NaCl, 20 m*M* imidazole pH 8.0). The protein was eluted in elution buffer (loading buffer plus 250 m*M* imidazole) and was subsequently subjected to TEV protease cleavage at a 1:100 mass ratio while dialyzing against loading buffer at 4°C overnight. The protein was then collected as the flowthrough from a second subtracting Ni–NTA column. The protein was further purified to homogeneity by size-exclusion chromatography using a buffer consisting of 20 m*M* HEPES Na pH 7.2, 150 m*M* NaCl. The protein was concentrated to 12 mg ml^−1^ for crystallization. Hsp90α_293–554 (Hsp90αM), Hsp90α_433–696 (Hsp90αC+) and Hsp90α_23–699_E47A (FL Hsp90α) were cloned, expressed and purified using the same procedure as for Hsp90αMC. This version of full-length (FL) Hsp90α was constructed to favor the open conformation of Hsp90, which is the conformation observed for the Hsp90αMC construct. The E47A mutation inhibits ATP hydrolysis but not its binding (Grenert *et al.*, 1999 ▸), while the Δ23 N-terminal mutation does not inhibit ATP binding but inhibits ATP hydrolysis and ATP-induced dimerization of the N-terminal domain (Richter *et al.*, 2002 ▸) and C-terminal truncation eliminates the unstructured C-terminal tail.

### 2.2. Crystallization, data collection and structural determination

Hsp90αMC was crystallized in the condition 0.1 *M* sodium acetate pH 6.0, 12%(*v*/*v*) PEG 3350 using the sitting-drop vapor-diffusion method at 20°C. To obtain MDCC {7-diethylamino-3-[*N*-(2-maleimidoethyl)carbamoyl]coumarin}-labeled protein, 0.2 m*M* Hsp90αMC was mixed with 0.5 m*M* MDCC (Sigma) and incubated on ice for 30 min. The sample was then centrifuged at 14 000 rev min^−1^ at 4°C to remove the precipitate that formed and the supernatant was used for crystallization. MDCC–Hsp90αMC crystals were obtained in a condition consisting of 0.1 *M* succinic acid pH 6.5, 12%(*v*/*v*) PEG 3350, 20 m*M* calcium chloride. 20% glycerol was added to the mother liquor as a cryoprotectant before flash-cooling in liquid nitrogen.

All data were collected on beamline 19-ID at the Advanced Photon Source (APS), Argonne National Laboratory. Diffraction data were processed using *HKL*-3000 (Minor *et al.*, 2006 ▸). The structure was solved by the molecular-replacement method using *Phaser* (McCoy *et al.*, 2007 ▸) with PDB entry 3q6m as the template (Lee *et al.*, 2011 ▸). *Phenix* was used for refinement (Liebschner *et al.*, 2019 ▸). Translation, libration and screw-rotation displacement groups used in refinement were defined by the *TLSMD* server (Painter & Merritt, 2006 ▸). *Coot* was used for iterative manual structural building (Emsley *et al.*, 2010 ▸). The final *R*_work_ and *R*_free_ for the refined models were 21.6% and 27.2%, respectively. The current model has good geometry and refinement statistics (Table 1 ▸). All molecular-graphics figures were generated with *PyMOL* (Schrödinger). The structure was deposited in the Protein Data Bank with accession code 7ry1.

### 2.3. Dynamic light scattering

The particle and molecular sizes of protein with or without inhibitors were analyzed using a Malvern Zetasizer DLS instrument. 5 µl of each of the proteins, 3 mg ml^−1^ Hsp90αMC in 20 m*M* HEPES pH 7.2, 150 m*M* NaCl and 3 mg ml^−1^ Hsp90αMC incubated with 0.5 m*M* inhibitors (chlorobiocin, derrubone, coumermycin A1 or MDCC) on ice for 30 min in 20 m*M* HEPES pH 7.2, 150 m*M* NaCl, 5%(*v*/*v*) DMSO, were loaded into the cuvette for DLS measurements at 25°C. The estimated molecular mass from DLS was used to analyze the oligomeric state of each protein with or without incubation with inhibitors. For the Hsp90αM protein, 2.1 mg ml^−1^ Hsp90αM was incubated with the inhibitors using the same procedure. Hsp90αC+ at 1.0 mg ml^−1^ and FL Hsp90α at 2.1 mg ml^−1^ were also used in DLS measurements with the same procedure as described above. All of the samples were centrifuged at high speed (14 000 rev min^−1^ for 15 min at 4°C) to remove precipitate before loading into the cuvette.

### 2.4. Fluorescence competition binding assay

Fluorescence measurements were performed in black 96-well microtiter plates (Corning #3650) using a Biotek Synergy H1 plate reader. MDCC fluorescence was excited at 419 nm and emission was recorded at 474 nm. For the competition binding studies, the Hsp90 allosteric inhibitors dissolved in DMSO were added to Hsp90αMC protein (2 µ*M*) in fluorescence assay buffer [20 m*M* HEPES pH 7.2, 150 m*M* NaCl, 5%(*v*/*v*) DMSO] to a final concentration of 50 µ*M* and incubated at room temperature for 3 h. MDCC was subsequently added to give final concentrations of 1 µ*M* Hsp90αMC and 1 µ*M* MDCC in a total volume of 200 µl. Fluorescence recording was started immediately and continued every 5 min for 1.5 h. The same method and procedure were used for the competition assay of Hsp90αM. Due to the aggregation effect of MDCC on Hsp90αC+ and FL Hsp90α, neither of these proteins were used in fluorescence competition studies.

### 2.5. Molecular docking

Based on the MDCC–Hsp90αMC structure, derrubone and chlorobiocin were respectively manually docked into the Hsp90 protein to replace MDCC in *Coot*. The *N*-ethylmaleimide hydrophobic binding area of MDCC was occupied by the 3-methylbut-2-enyl group of the inhibitors. The isoflavone and coumarin ring binding sites of these inhibitors are close to each other.

## 3. Results

### 3.1. Hexameric structure of MDCC–Hsp90αMC

In our initial studies to identify the binding site for Hsp90α MD and CTD inhibitors, Hsp90αMC (residues 293–699) was expressed and purified (Supplementary Fig. S1) and subjected to co-crystallization screening using numerous conditions together with different putative allosteric inhibitors, including derrubone, chlorobiocin, coumermycin A1, celastrol and garcinol. Unfortunately, no inhibitors were observed in our co-crystal structures.

**Figure 1:**
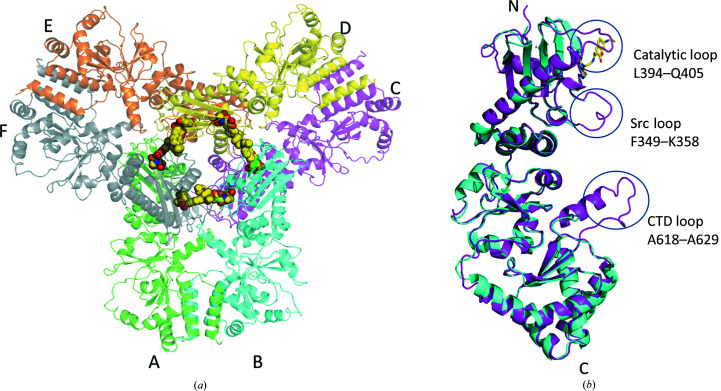
The hexameric structure of MDCC–Hsp90αMC. (*a*) The hexamer is shown in cartoon representation and is colored green (chain *A*), cyan (chain *B*), magenta (chain *C*), yellow (chain *D*), orange (chain *E*) and gray (chain *F*). MDCC molecules are shown as spheres and are located at the hexamer interface. (*b*) Structural alignment of the monomer of chain *C* (magenta) in MDCC–Hsp90αMC (MDCC is shown as yellow sticks) with that in the apo structure (PDB entry 3q6n, cyan). The three ordered loops, the catalytic loop, Src loop and CTD loop, are indicated.

Recently, we identified a putative coumarin derivative binding site within the hinge region between the MD and CTD of Hsp90α (Matts, Dixit *et al.*, 2011 ▸). Two cysteine residues (Cys572 and Cys598) are located near the identified site. Therefore, we attempted to co-crystallize Hsp90αMC with a coumarin derivative, 7-diethylamino-3-[*N*-(2-maleimidoethyl)carbamoyl]coumarin (MDCC), which contains the coumarin ring that is shared among many previously characterized Hsp90MC inhibitors. MDCC contains an *N*-ethylmaleimide moiety that could form a covalent link to exposed reduced cysteines (Kusuma *et al.*, 2014 ▸; Kunzelmann & Webb, 2009 ▸), which might aid in stabilizing the inhibitor binding in an affinity-labeling manner. The MDCC–Hsp90αMC structure was determined at 3.5 Å resolution. Unexpectedly, we observed MDCC to only be covalently linked to Cys374 in the MD, although there are six additional cysteines in Hsp90αMC (Cys420, Cys481, Cys529, Cys572, Cys597 and Cys598; Supplementary Fig. S2). The MDCC–Hsp90αMC structure contains three molecules in the asymmetric unit, which further form a hexamer through crystallographic symmetry (Fig. 1 ▸ *a*). This hexameric association of the MDCC–Hsp90αMC complex structure is similar to that observed in the apo Hsp90αMC structure (PDB entry 3q6n; Lee *et al.*, 2011 ▸), with molecule *A* in the two structures having a root-mean-square deviation (r.m.s.d.) of 0.86 Å over 393 aligned C^α^ atoms. The current structure differs from the previous structure in three loop regions that become ordered upon MDCC binding. These include two loops in the MD, loop 394-LPLNISREMLQQ-405 (often referred to as the catalytic loop; Cunningham *et al.*, 2012 ▸; Meyer *et al.*, 2003 ▸), which contains the Arg400 residue that interacts with the γ-phosphate of ATP and stabilizes the closed conformation of Hsp90 that is required for ATP hydrolysis (Cunningham *et al.*, 2012 ▸), and loop 349-FDLFENRKKK-358, which is often referred to as the Src loop (Shiau *et al.*, 2006 ▸). In addition, the CTD loop 618-ALRDNSTMGYMA-629 following the CTD amphipathic helix is also ordered (Fig. 1 ▸ *b*). The main chains and some of the side chains in these three regions could be clearly traced in the current structure, while they were all disordered in previous human Hsp90αMC structures (Lee *et al.*, 2011 ▸). The structure suggests that the Src loop and catalytic loop in the MD and the CTD loop could play an essential role in Hsp90 oligomerization and inhibitor binding.

### 3.2. The hydrophobic binding pocket for MDCC

**Figure 2:**
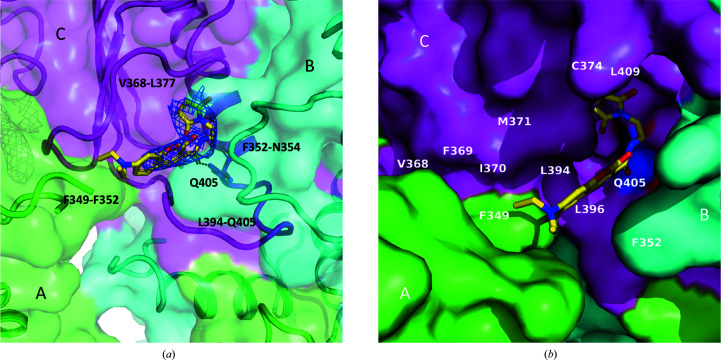
MDCC is bound in a composite hydrophobic binding pocket at the hexamer interface. (*a*) MDCC is sandwiched between loop Val368–Leu377 and the catalytic loop (Leu394–Gln405) in molecule *C*, as well as part of two Src loops (Phe349–Phe352 and Phe352–Asn354) from molecules *A* and *B*. MDCC is shown in stick representation and is enveloped by 2*mF*_o_ − *DF*_c_ electron-density maps. (*b*) MDCC binding pocket. Key residues lining the pocket are indicated. The coloring scheme for the protomers is the same as in Fig. 1 ▸(*a*).

In the current structure, each protomer binds an MDCC molecule in the MD and a total of six MDCC molecules are located in the Hsp90αMC hexamer interface (Fig. 1 ▸). The electron densities for the head part of MDCC (*N*-ethyl­maleimide; NEM) and the coumarin core are clearly visible, while the diethylamino tail is disordered (Fig. 2 ▸ *a*). The NEM head is found to only be covalently linked to Cys374 and not to any of other six cysteines in the complex structure (Figs. 1 ▸ *a* and 2 ▸ *b*).

Each of the MDCC molecules is located inside a composite hydrophobic pocket at the hexamer interface, contacting Hsp90αMC mainly through hydrophobic interactions. The hydrophobic MDCC-binding pocket is constructed mainly by four loops from three neighboring Hsp90αMC molecules. Each pocket includes parts of the Src-loop regions (349-FDLF-352 from one protomer and 352-FEN-354 from the other in the Hsp90αMC homodimer), as well as the loop region 368-VFIMDNCEEL-377 and the catalytic loop 394-LPLNISREMLQQ-405 from a third molecule (Fig. 2 ▸ *a*). The hydrophobic loop 368-VFIM-371 and the hydrophobic residue Leu396 located in the catalytic loop of molecule *C*, as well as the aromatic residues Phe349 in the Src loop of molecule *A* and Phe352 in the Src loop of molecule *B*, form the hydrophobic binding pocket for the coumarin core of MDCC (Fig. 2 ▸ *b*). The NEM head of MDCC is also surrounded by hydrophobic residues, including Ile370, Met371, Cys374, Leu394, Leu396 and Leu409, in each protomer (Fig. 2 ▸ *b*). Besides these hydrophobic interactions, the carbonyl group of MDCC forms a hydrogen bond to Gln405 at the end of the catalytic loop in the MD (Fig. 2 ▸ *a* and Supplementary Fig. S3).

### 3.3. MDCC binding blocks the cochaperone/client binding sites on the Src loop

**Figure 3:**
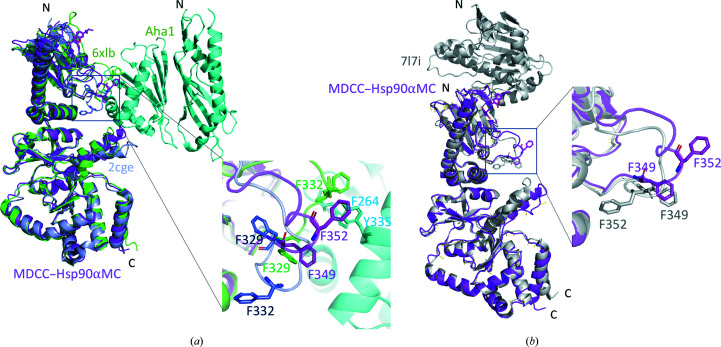
Conformational changes of the Src loop induced by MDCC binding. (*a*) Structural superimposition of MDCC–Hsp90αMC (magenta), yeast Hsc82 (light blue; PDB entry 2cge) and the yeast Hsc82–Aha1 complex (Hsc82, green; Aha1, cyan; PDB entry 6xlb). (*b*) Structural superimposition of MDCC–Hsp90αMC (magenta) and closed-state full-length human Hsp90α (gray; PDB entry 7l7j). The insets detail the Src-loop conformations, with the two Phe residues shown in stick representation.

The Src-loop region is disordered in apo human Hsp90αMC hexamer structures (Lee *et al.*, 2011 ▸) and is stabilized by MDCC binding in the current structure. The conformation of the Src loop can also be clearly traced in the previously reported yeast Hsp90MC hexamer structure (PDB entry 2cge; Ali *et al.*, 2006 ▸) and the yeast Hsc82–Aha1 complex structure (PDB entry 6xlb; Liu *et al.*, 2020 ▸), as well as in full-length human Hsp90 structures complexed with cochaperone and client proteins, such as the Hsp90β–Cdc37–Cdk4 (PDB entry 5fwk), Hsp90α–FKBP51–p23 (PDB entry 7l7i) and Hsp90α–p23 (PDB entry 7l7j; Lee *et al.*, 2021 ▸; Verba *et al.*, 2016 ▸) complexes. Structure alignment of the Src loops in these structures revealed large conformational changes (Fig. 3 ▸). When compared with the yeast Hsp82 hexamer structure, two aromatic residues in the Src loop (Phe349 and Phe352 in human Hsp90 and Phe329 and Phe332 in Hsp82) display opposite orientations. They are rotated outwards in the current structure and are involved in hydrophobic interactions with the coumarin core of MDCC (Figs. 2 ▸ *b* and 3 ▸ *a*). In contrast, the corresponding Phe residues in Hsp82 turn totally inwards. Strikingly, when in complex with Aha1 (6xlb), both Phe side chains in Hsc82 are rotated outwards in similar orientations as in the current structure, contacting Phe264 and Tyr335 of Aha1 through hydrophobic interactions (Fig. 3 ▸ *a*). In the closed-state, full-length Hsp90α–p23 complex (PDB entry 7l7j), Phe349 adopts the same outwards direction as in the current structure, while Phe352 adopts an opposite orientation (Fig. 3 ▸ *b*), displaying the same conformations as in the structures of the Hsp90β–Cdc37–Cdk4 and Hsp90α–FKBP51–p23 complexes (not shown). Our structural comparison suggests that MDCC binding could block the binding of the Src loop by cochaperone and client proteins.

### 3.4. MDCC binding reorients the catalytic loop

**Figure 4:**
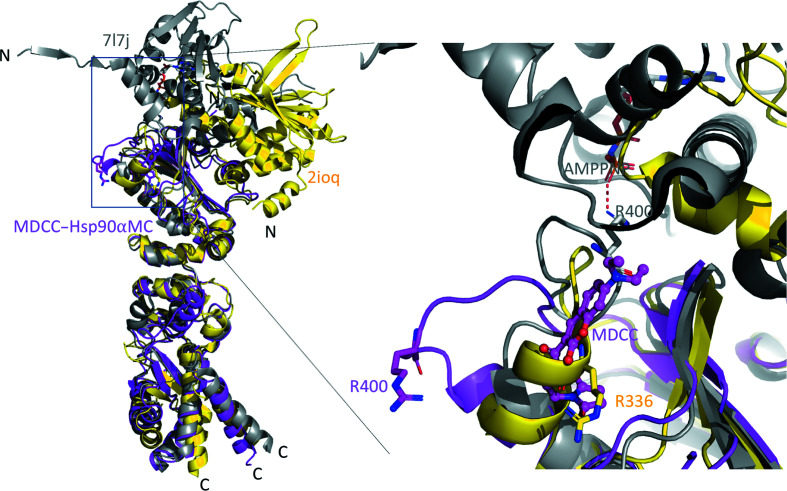
Different conformations of the catalytic loop and Arg400. Structural superimposition of MDCC–Hsp90αMC (magenta), the protomer in the *E. coli* Hsp90/HTPG open-state dimer (without ATP binding, yellow; PDB entry 2ioq) and the protomer in the human Hsp90α closed-state dimer (with ATP analog bound, gray; PDB entry 7l7j). The inset details the various conformations of the catalytic loop and Arg400. MDCC and Arg400 are shown in stick representation.

The conformation of the catalytic loop is flexible in Hsp90α MD. It has been shown in the ATP-bound closed-state human Hsp90 dimer structure that the catalytic residue Arg400 (Arg380 in yeast Hsp82 and Arg336 in *E. coli* HTPG) displays an open ‘active’ state that forms a hydrogen bond to the γ-phosphate of the bound ATP in the NTD (Prodromou, 2016 ▸; Ali *et al.*, 2006 ▸; Lee *et al.*, 2021 ▸). In contrast, in the open state of the human Hsp90 dimer structure without bound ATP Arg400 is re­oriented and is held in a closed ‘inactive’ state, *i.e.* a conformation that is incapable of hydrogen bonding to ATP (Shiau *et al.*, 2006 ▸). To analyze the catalytic loop conformation in our current structure, we compare it with both the open-state dimer structure (without ATP binding) of *E. coli* Hsp90/HTPG (PDB entry 2ioq) and the closed-state dimer structure (with ATP analog binding) of human Hsp90α (PDB entry 7l7j). Structural superimposition showed that the MDCC in our current structure overlaps with the position of Arg336 in the catalytic loop of *E. coli* HTPG in the open-state dimer, which positions Arg400 in an opposite orientation with respect to the ATP-bound closed-state dimer structure of human Hsp90α (Fig. 4 ▸). In addition, Gln405 at the end of the catalytic loop interacts with MDCC via a hydrogen bond to the carbonyl group of MDCC and via hydrophobic interactions between Leu396 and the coumarin core of MDCC (Fig. 2 ▸ and Supplementary Fig. S3). Our structure suggests that MDCC binding locks Arg400 in a conformation which cannot stabilize the γ-phosphate of ATP during hydrolysis.

### 3.5. MDCC binding strengthens Hsp90αMC hexamer association

**Figure 5:**
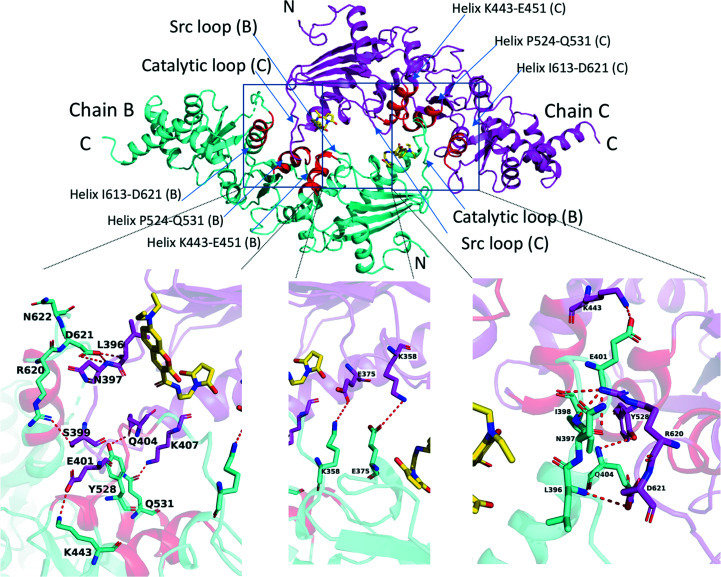
The hexamer interface involving the catalytic loop from one protomer (chain *C*, magenta) of a homodimer contacting the MC domain in a neighboring molecule (chain *B*, cyan). The catalytic loops are indicated and the three helices in the MC domain are highlighted in red. Each inset details the interactions between the catalytic loop and the MC domain.

**Figure 6:**
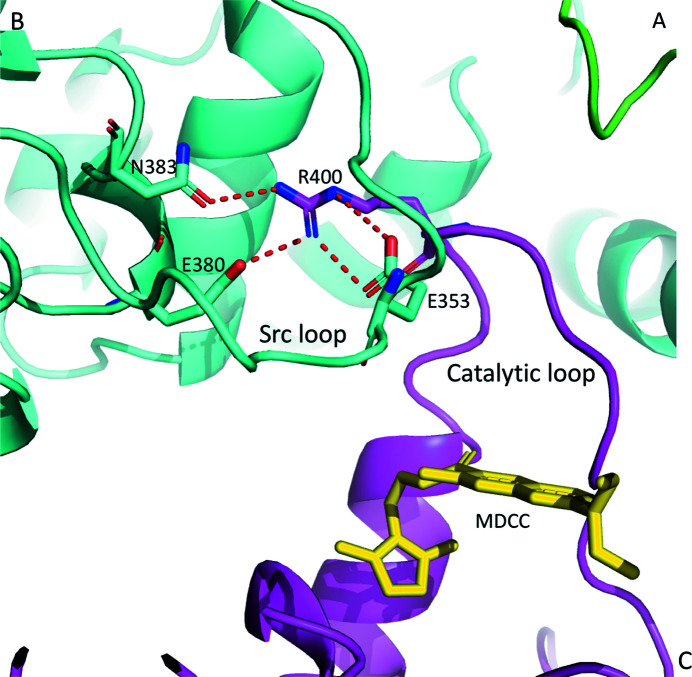
Arg400 in the catalytic loop tethers the Src loop at the hexamer interface. The catalytic loop of chain *C* (magenta) interacts with the Src loop of chain *B* (cyan). Hydrogen bonds are shown as red dashed lines and MDCC is shown as yellow sticks.

**Figure 7:**
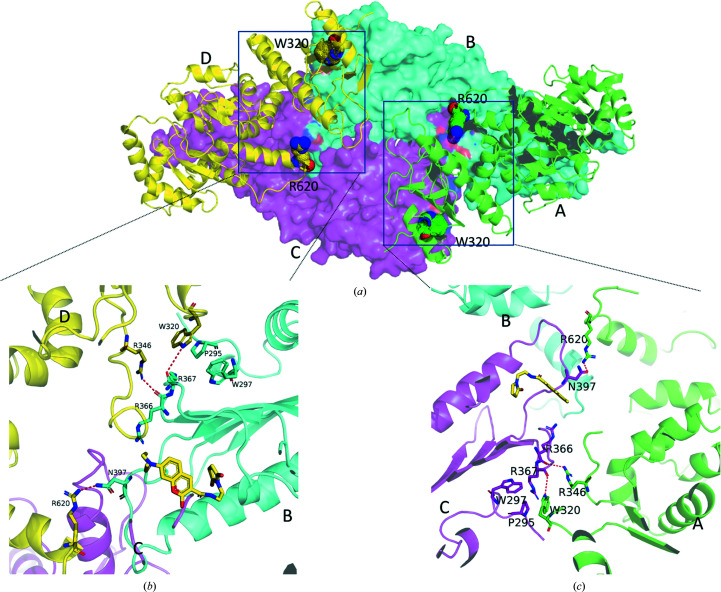
Hexamer interface involving Trp320 in the MD and Arg620 in the CTD loop. (*a*) Chain *A* (green) and chain *D* (yellow) are shown as cartoons, and chain *B* and chain *C* are shown as surfaces. The coloring scheme is the same as in Fig. 1 ▸(*a*). Trp320 and Arg620 are shown as spheres. The insets (*b*, *c*) detail the molecular interactions involving Trp320 and Arg620. Hydrogen bonds are shown as red dashed lines.

The hexameric structure is comprised of three Hsp90αMC homodimers (Fig. 1 ▸ *a*). Three regions are involved in hexa­merization. The first region at the hexamer interface involves the catalytic loops and Src loops. The binding of MDCC stabilizes these loops, making them clearly visible. The catalytic loop of each protomer in the Hsp90αMC homodimer intrudes into the MC domain of a third molecule from another Hsp90αMC dimer, in turn forming an interdigitated homohexamer (Figs. 1 ▸ *a* and 5 ▸). Specifically, the catalytic loop of one protomer interacts with three α-helices (Lys443–Glu451, Pro524–Gln531 and Ile613–Asp621) in the MD and CTD of another via hydrogen bonding. The main-chain amide of Leu396 and the side chain of Asn397 in the catalytic loop are hydrogen bonded to Asp621 from the neighboring CTD loop. The side chains of Ser399, Glu401, Gln404 and Lys407 in the catalytic loop are hydrogen bonded to Arg620, Lys443, Tyr528 and Gln531, respectively, in the other molecule (Fig. 5 ▸). In addition, each of the ordered catalytic loops from the homodimers contacts the ordered Src loop in a third protomer, involving a salt bridge (Arg400–Glu353; Fig. 6 ▸). Arg400 additionally forms hydrogen bonds to the main-chain carbonyl of Glu380 and the side chain of Asn383 from the third molecule (Fig. 6 ▸). Lys358 in the Src loop also forms a salt bridge with Glu375 in the neighboring molecule (Fig. 5 ▸). The second region of the hexamer interface is at the N-terminus of the protein and involves Trp320, which bridges two neighboring molecules. While Trp320 from one protomer of the homodimers forms hydrophobic interactions with Pro295 from the third protomer, its indole ring also forms a hydrogen bond to the main-chain carbonyl of Arg367 from the third protomer (Fig. 7 ▸). These interactions were also observed in previously published apo Hsp90MC structures (Lee *et al.*, 2011 ▸). The third region in the hexamer interface involves Arg620 in the CTD loop. Arg620 on one protomer is hydrogen-bonded to Asn397 on the catalytic loop of another neighboring protomer at the hexamer interface (Fig. 7 ▸). Except for the region of the Trp320 interactions, all other regions in these interfaces are disordered in apo Hsp90αMC structures (Lee *et al.*, 2011 ▸), which suggests that MDCC binding strengthens Hsp90 hexamerization.

### 3.6. MC-domain inhibitors stimulate Hsp90 oligomerization in solution

**Figure 8:**
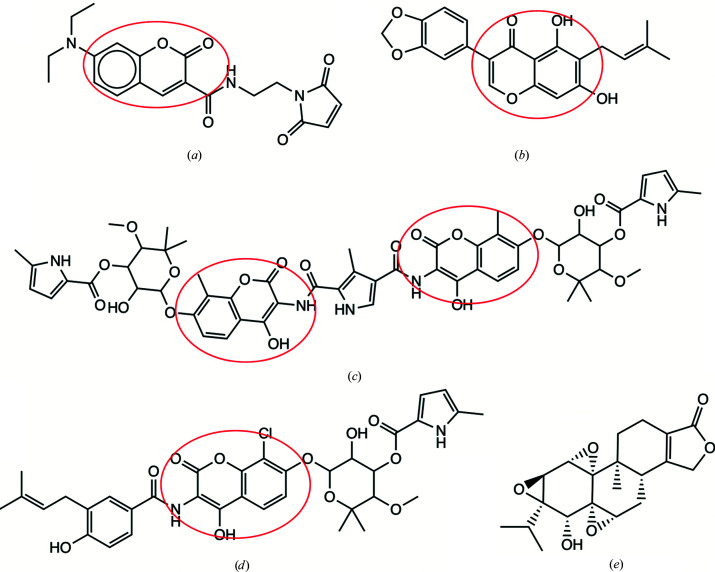
Structures of MDCC and the inhibitors. (*a*) MDCC. (*b*) Derrubone. (*c*) Coumermycin A1. (*d*) Chlorobiocin. (*e*) Triptolide. The red circles indicate the coumarin core.

The current complex structure suggests that the binding of an MD/CTD inhibitor could stabilize the Hsp90αMC hexamer interface. We therefore analyzed the oligomeric states of Hsp90αMC and Hsp90αM in solution by gel filtration (SEC) and dynamic light scattering (DLS) with and without allosteric inhibitors. Hsp90αMC purified mainly as a dimer in solution by SEC, while Hsp90αM purified mainly as a monomer in solution by SEC (Supplementary Figs. S1 and S4). However, the purified dimeric Hsp90αMC protein displayed a strong tendency to oligomerize into a hexameric state over time as observed from DLS. Interestingly, we found that in the presence of known allosteric inhibitors, including derrubone, chlorobiocin and coumermycin A1, the oligomerization of dimeric Hsp90αMC into its hexameric state was greatly accelerated and stabilized (Supplementary Table S1, Fig. 8 ▸). In contrast, neither chlorobiocin nor coumermycin A affected the oligomeric state of Hsp90αM, although derrubone tends to cause the MD to form a dimer in solution. SEC and DLS indicated that the CTD was a tetramer (Supplementary Fig. S5) and FL Hsp90α was a hexamer (Supplementary Fig. S6) in solution. However neither protein was stable in the presence of MDCC and inhibitors, which led to the formation of large aggregates (Supplementary Table S1).

### 3.7. MDCC shares a common binding site with other coumarin-derivative inhibitors

**Figure 9:**
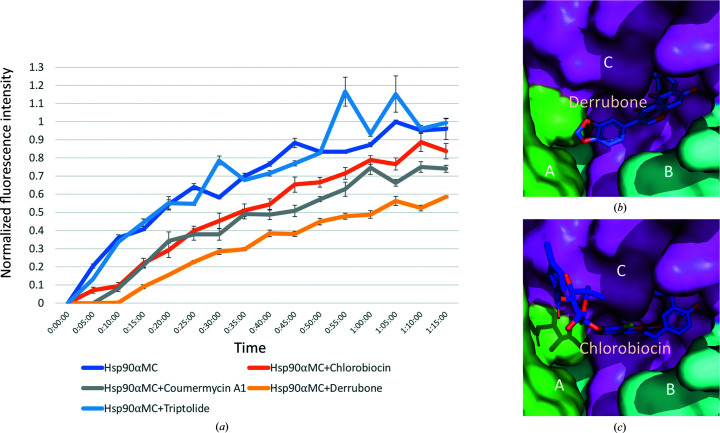
(*a*) Fluorescence-based competition assay. Various coumarin core-containing inhibitors were pre-incubated with Hsp90αMC before adding MDCC, the fluorescence intensity of which was monitored over time. Models of derrubone (*b*) and chlorobiocin (*c*) bound in Hsp90αMC (chain *A*, green; chain *B*, cyan; chain *C*, magenta).

To evaluate whether the hydrophobic pocket of MDCC represents a common allosteric binding site for other Hsp90 coumarin core-containing inhibitors, we performed a competitive binding assay by monitoring the fluorescence signal of MDCC. The quantum yield of the coumarin ring in MDCC is dependent on the local environment in which it is bound (Haugland, 1996 ▸; Schauer-Vukasinovic *et al.*, 1997 ▸). Labeling Cys residues, which leaves MDCC completely exposed to solvent, causes no change in its intrinsic fluorescence (Goodey *et al.*, 2011 ▸). In contrast, the quantum yield of MDCC would markedly increase upon its sequestration in the hydrophobic environment of the Hsp90α hexamer. The MDCC fluorescence signal was measured using excitation at 419 nm and emission at 474 nm (Case *et al.*, 2019 ▸; Brune *et al.*, 1998 ▸; Kunzelmann & Webb, 2009 ▸). Chlorobiocin and coumermycin A1, which both contain a coumarin scaffold, and derrubone, which contains the related isoflavone scaffold, are considered to be Hsp90 allosteric inhibitors (Hadden *et al.*, 2007 ▸; Marcu, Schulte *et al.*, 2000 ▸; Cele *et al.*, 2016 ▸; Burlison & Blagg, 2006 ▸; Hastings *et al.*, 2008 ▸). DMSO and triptolide were also tested as negative controls in the assay (Zhang *et al.*, 2018 ▸). Coumarin inhibitors were pre-incubated with Hsp90αMC for 3 h before MDCC was added and the fluorescence intensity was measured immediately. The results showed that in the presence of derrubone, chlorobiocin or coumermycin A1 the MDCC fluorescence signal was significantly delayed and weakened, while the presence of either DMSO or triptolide showed no effect (Fig. 9 ▸ *a*). In contrast, we did not observe similar competition effects of the inhibitors with MDCC for Hsp90αM (Supplementary Fig. S7). These data are consistent with our structural observation that the MDCC binding pocket is a composite binding site at the hexamer interface and is stabilized by both the MD and CTD, and that the coumarin/isoflavone core contained in the inhibitors could compete with MDCC for binding to a common site on Hsp90.

We next attempted to build models of the coumarin-related Hsp90 inhibitors binding to the MDCC binding site. Our modeling shows that both derrubone and chlorobiocin could fit into the MDCC binding pocket at the hexamer interface (Fig. 9 ▸ *b* and 9 ▸ *c*).

## 4. Discussion

Small-molecule compounds that inhibit Hsp90 are selectively cytotoxic to transformed cells and are less toxic to normal cells (Kamal *et al.*, 2003 ▸). Reflecting this promise, more than 24 clinical trials have been conducted or initiated investigating Hsp90 NTD inhibitors for the treatment of cancer (Jhaveri *et al.*, 2014 ▸; Garcia-Carbonero *et al.*, 2013 ▸; Zagouri *et al.*, 2013 ▸; Neckers & Workman, 2012 ▸). Some of the trials have been very encouraging, but others have been disappointing because they revealed dose-limiting clinical complications and toxicities. The most significant shortcoming of Hsp90 NTD inhibitors is their induction of the HSR (Garcia-Carbonero *et al.*, 2013 ▸; Jhaveri *et al.*, 2014 ▸; Neckers & Workman, 2012 ▸). This response represents an anti-apoptotic cell-survival response that is widely believed to lower the ability of Hsp90 inhibitors to induce tumor cell death (Neckers & Workman, 2012 ▸; Trepel *et al.*, 2010 ▸; Brandt & Blagg, 2009 ▸). This also creates difficulties in dosing schedules in clinical trials (Neckers & Workman, 2012 ▸). In contrast to the N-terminal ATP-binding pocket of Hsp90, however, targeting the MD and CTD of Hsp90 is an alternative strategy for allosteric inhibition (Brandt & Blagg, 2009 ▸; Donnelly & Blagg, 2008 ▸). Unlike NTD inhibitors, these allosteric inhibitors, such as novobiocin, block Hsp90 function without concomitant induction of the HSR (Matts, Brandt *et al.*, 2011 ▸), which is pro-survival and is responsible for the difficulty observed in dosing schedules. It is this absence of induction of the HSR that suggests that allosteric Hsp90 inhibitors may represent superior chemotherapeutic agents for the treatment of cancer. The development of Hsp90 allosteric inhibitors is greatly hindered by the lack of any experimentally determined co-structures. The MDCC-bound Hsp90αMC structure therefore provides a first look at this class. We observed precipitation of the protein after incubation with MDCC and only used the supernatant for crystallization (see Section 2). It is likely that MDCC conjugation to any other sites caused the disruption of the folding of the protein, leading to nonspecific aggregation and precipitation of the protein, and probably accounts for the increase in the background fluorescence that is seen at longer incubation times in the binding competition experiments. Therefore, the unique MDCC binding site identified at Cys374 in the MD could be biologically relevant. This is further supported by the fluorescence-based competition studies with coumarin core-containing inhibitors, although we could not exclude the possibility that chlorobiocin and derrubone could also bind to another site on the protein and allosterically affect the binding of MDCC. MDCC is located at a composite hydrophobic binding pocket at the hexamer interface. The binding of MDCC induced the ordering of three regions in Hsp90, including the catalytic loop, the Src loop and a CTD loop (Supplementary Fig. S8), which adopt unique conformations and contribute to Hsp90 hexamer association (Supplementary Fig. S9). The structure suggests that MDCC binding locks Hsp90 into an inactive hexamer, with one key residue Arg400 oriented away from the ATP-binding pocket so that the stabilization of the NTD and MD that is required for optimal ATP hydrolysis is not possible (Cunningham *et al.*, 2012 ▸). In addition, both the CTD loop and the Src loop are also shielded from client binding. Our structure provides mechanistic insights into the function of Hsp90 and its inhibition by coumarin scaffold-containing allosteric inhibitors.

While primarily a homodimer, Hsp90 is also present in different oligomeric states both in cultured cells (Nemoto & Sato, 1998 ▸) and in purified *in vitro* systems (Jakob & Buchner, 1994 ▸; Minami *et al.*, 1991 ▸). The dimer–oligomer equilibrium is shifted towards the oligomerized state upon heat shock and in the presence of non-ionic detergents, divalent cations and higher Hsp90 concentrations, but the shift is inhibited in the presence of nucleotides and Hsp90 NTD inhibitors (for example geldanamycin; Chadli *et al.*, 1999 ▸; Jakob *et al.*, 1995 ▸; Minami *et al.*, 1993 ▸; Yonehara *et al.*, 1996 ▸; Garnier *et al.*, 1998 ▸, 2002 ▸). During the self-association process, the Hsp90 homodimer is the building block to form higher ordered oligomers, including tetramers, hexamers and dodecamers. The tetramers represent an intermediate state between dimers and hexamers. The equilibrium of Hsp90 dimers and oligomers is postulated to be important for modulating Hsp90 function (Nemoto *et al.*, 2001 ▸; Yonehara *et al.*, 1996 ▸; Buchner, 1999 ▸; Jakob & Buchner, 1994 ▸; Freeman & Morimoto, 1996 ▸; Wiech *et al.*, 1992 ▸; Minami *et al.*, 2001 ▸). The ‘holdase’ function of oligomeric Hsp90 prevents the irreversible aggregation of denatured proteins and does not require the presence of ATP (Yonehara *et al.*, 1996 ▸). In plants, oligomers of Hsp90 display more ‘holdase’ chaperone activity than dimers (Cha *et al.*, 2013 ▸). However, to carry out the ATP-dependent folding (‘foldase’) activity, the oligomers of Hsp90 need to dissociate into dimers that are capable of undergoing conformational changes driven by the binding and hydrolysis of ATP (Schopf *et al.*, 2017 ▸). In the cell, the transition between the oligomeric and dimeric states is likely to be regulated by the binding of ATP (Chadli *et al.*, 1999 ▸), post-translational modifications, for example sumoylation (Mollapour *et al.*, 2014 ▸) or phosphorylation (Xu *et al.*, 2012 ▸, 2019 ▸), and/or cochaperones, such as Hsp70, Aha1 and p23 (Wolmarans *et al.*, 2016 ▸; Lepvrier *et al.*, 2018 ▸).

**Figure 10:**
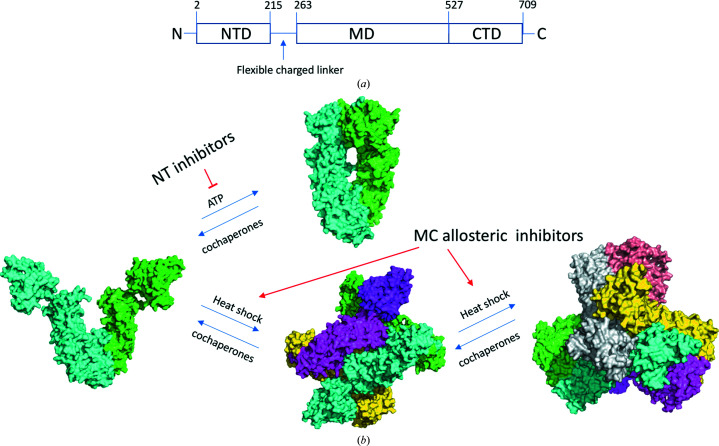
Oligomeric states of FL Hsp90. (*a*) Domain architecture (NTD, MD and CTD) of Hsp90. (*b*) Model of Hsp90 oligomerization and disassembly.

Based on the current structure, we propose a hexameric FL structure model of Hsp90 that is in a stable open conformation in each dimer, which prevents the NTD of each dimer from forming the closed state (Fig. 10 ▸). Our structural model is different from the hexameric model described in a previous report, which had two open-state dimers and one semi-open dimer in the hexamer (Lepvrier *et al.*, 2018 ▸). Since the charged linker connecting the NTD and MD is flexible, we made the model based on the hexameric structure of the MC domain in a similar way to that described previously (Lee *et al.*, 2011 ▸). Our data suggest that the binding of MC inhibitors to Hsp90αMC stabilizes Hsp90 in its hexameric state, switching the equilibrium away from the dimeric state.

The structure of the MDCC–Hsp90αMC hexamer indicates that the regions involved in critical functions (for example ATP hydrolysis and client binding) that are required for Hsp90-dependent protein folding are sequestered at the hexamer interface, including the catalytic loop, the Src loop and the CTD loop: the catalytic loop is essential for ATP hydrolysis, while the Src-loop and CTD-loop regions are involved in client binding. The catalytic loop of each protomer within the current structure is enveloped by a third molecule, which stabilizes the homodimer block of Hsp90 in the open state with the catalytic loop in an inactive orientation that differs from that of the closed conformational state of Hsp90 during ATP hydrolysis and client binding (Meyer *et al.*, 2003 ▸; Xu *et al.*, 2012 ▸). Specifically, the catalytic loop is tethered by Arg620 in the CTD loop and other residues in the MD, leading to a conformation of Arg400 that is not suitable for stabilizing the γ-phosphate of ATP for hydrolysis and preventing the Src loop from engaging in the binding of client proteins (Figs. 3 ▸ and 4 ▸). However, via its diethylamino tail and through hydrophobic interactions, MDCC stabilizes the Src loop in a buried conformation. A recently published structure in the PDB (PDB entry 6xlb) suggests that the C-terminal domain of Aha1 could capture dimeric Hsp90 by binding to Phe352 in the Src loop (Liu *et al.*, 2020 ▸). However, MDCC binding locks Phe352 in a sequestered conformation that is not accessible by Aha1 (Fig. 10 ▸). The Src loop was found to be key to the binding of certain clients (Verba *et al.*, 2016 ▸); therefore, its sequestration by MDCC binding could prevent the folding of these client proteins. Consistent with our observation, the Src loop was also found to bind other Hsp90 allosteric binding inhibitors targeting the MD that were identified by NMR (Zhou *et al.*, 2020 ▸).

With the client-binding sites in the MC domain sequestered, how could hexameric FL Hsp90 have a ‘holdase’ function for selected client proteins? The formation of the hexameric state in the context of FL Hsp90 that contains the MC hexameric core described here would require that the NTD of Hsp90 become undocked from the MC domain of Hsp90. This is because residues Pro295 and Arg367 that are important for tethering the NTD and MD in the closed Hsp90 dimer structure (Lee *et al.*, 2021 ▸) are now buried at the MC hexamer interface and contact Trp320 instead. In fact, it has been demonstrated previously by single-molecule FRET studies that undocking and docking of the charged linker region between the NTD and the MD is in a rapid equilibrium, which has an important regulatory role that couples the arrangement of the Hsp90 NTD and MD to accessibility of the client-binding site on the NTD and client activation (Jahn *et al.*, 2014 ▸; López *et al.*, 2021 ▸) and regulates crosstalk between peptide binding to the NTD and ATP binding (Scheibel *et al.*, 1999 ▸; Young *et al.*, 1997 ▸). Therefore, the client-binding activity of the NTD (Scheibel *et al.*, 1998 ▸) and the anti-aggregation properties of the intrinsically unstructured acidic charged linker region and C-terminal tail of Hsp90 (Wayne & Bolon, 2010 ▸) could possibly account in part for the ‘holdase’ function of the hexamer structure of FL Hsp90.

Based on the recent cryo-EM structures of yeast Hsc82 in complex with Aha1 (PDB entries 6xlb and 6xlg; Liu *et al.*, 2020 ▸), we postulate the following model for the regulation of the equilibrium between the hexameric and dimeric states of Hsp90. The hexameric state is in equilibrium with the dimeric state. Upon the dissociation of hexamers into dimers, the initial binding of the N-terminal domain of Aha1 prevents the docking of the NTD of Hsp90 to the MD, and hence the C-terminal domain of Aha1 could capture the dimeric Hsp90 by binding to Phe352 in the Src loop (Liu *et al.*, 2020 ▸). Subsequently, upon ATP binding Hsp90 adopts a closed conformation with its NTD docked to the MD, and the CT domain of Aha1 is bound to Trp320 in the MD of Hsp90 (PDB entry 6xlh). Upon this reorientation of the domains, the conformation of the catalytic loop also reorients to coordinate the γ-phosphate of ATP and the client is captured in the hydrophobic pocket formed at the interface between the Src loop and CTD loop (Verba *et al.*, 2016 ▸; Noddings *et al.*, 2020 ▸; Wang *et al.*, 2020 ▸). The interaction of the CT domain of Aha1 with the Src-loop residue and Trp320 prevents any recruitment of the Hsp90 into the hexamer until the chaperone cycle is complete. Of note is the observation that the sumoylation of Hsp90α at Lys191 and phosphorylation of Tyr313 increase the binding affinity of Aha1 for Hsp90 and could play a role in modulating the hexamer–dimer equilibrium (Mollapour *et al.*, 2014 ▸; Xu *et al.*, 2012 ▸, 2019 ▸).

In summary, our data suggest that the hydrophobic binding pocket for the coumarin core of MDCC in the MD could be an allosteric inhibitor-binding site of Hsp90. This class of inhibitor could block the functions of Hsp90 by locking it into an inactive oligomeric state lacking either ATPase activity or productive client binding. Therefore, the MDCC binding pocket could serve as a new drug target for combating cancer and neurodegenerative and infectious diseases.

## Supplementary Material

PDB reference: Hsp90α MC domain covalently linked to MDCC, 7ry1

Supplementary Table and Figures. DOI: 10.1107/S2059798322002261/qh5073sup1.pdf

## Figures and Tables

**Table 1:** Crystallographic data-collection and refinement statistics for MDCC–Hsp90αMC Values in parentheses are for the highest resolution shell.

Data collection	
Beamline	19-ID, APS
Wavelength (Å)	0.97951
Space group	*C*222_1_
*a*, *b*, *c* (Å)	159.9, 311.9, 88.1
Resolution (Å)	50.00–3.50 (3.63–3.50)
Total reflections	176259
Unique reflections	27438 (2708)
Multiplicity	6.4 (6.4)
Completeness (%)	99.9 (100.0)
〈*I*/σ(*I*)〉	12.4 (1.2)
*R*_merge_ † (%)	14.8 (136.6)
CC_1/2_	0.993 (0.536)
Refinement statistics	
Resolution range used (Å)	47.4–3.52
No. of reflections used	27394
*R*_work_/*R*_free_ ‡ (%)	21.6/27.2
R.m.s.d., bond lengths (Å)	0.004
R.m.s.d., bond angles (°)	0.806
No. of atoms	
Protein	9949
Ligand	84
Water	0
Average *B* factors (Å^2^)	
Protein	141
Ligand	181
Ramachandran values	
Preferred regions (%)	95.2
Allowed regions (%)	4.8

†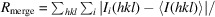 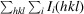 .

‡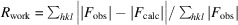 . *R*_free_ was calculated using 5% of data.
