# Supplementary material for: Crystal structure of the middle and C-terminal domains of Hsp90α labeled with a coumarin derivative reveals a potential allosteric binding site as a drug target
Source: Acta Crystallogr D Struct Biol. 2022 Apr 8;78(Pt 5):571–85. doi: 10.1107/S2059798322002261 (PMC9063849; doi:10.1107/S2059798322002261)
Supplement: Supplementary file 1 [file d-78-00571-sup1.pdf]

Acta Cryst D
STRUCTURAL
BIOLOGY

Volume 78 (2022)

Supporting information for article:

Crystal structure of the middle and C-terminal domains of Hsp90α labeled with a coumarin derivative reveals a potential allosteric binding site as a drug target

Shuxia Peng, Jeff Woodruff, Prabhat Kumar Pathak, Robert L. Matts and Junpeng Deng

Table S1 DLS analysis of the oligomeric states of Hsp90αMC, Hsp90αM, Hsp90αC, and Hsp90α FL proteins with inhibitors

Protein	+	Z-Ave (nm)	MW (kDa)	Oligomeric state
Hsp90αMC	Buffer	11.8+/-3.2	213.3+/-58.8	Tetramer (Dimer-Tetramer-Hexamer)
	DMSO	11.8+/-4.4	216+/-80.2	Tetramer (Dimer-Tetramer-Hexamer)
	Chlorobiocin	27.1+/-13.4	1500+/-745.5	Oligomer
	Coumermycin A1	16.8+/-4.9	452.3+/-137.8	Hexamer
	Derrubone	13.8+/-4.6	308.3+/-103.8	Hexamer
	MDCC	13.5+/-3.8	292.7+/-82.3	Hexamer
Hsp90αM	Buffer	6.4+/-1.6	51.8+/-13.3	Monomer
	DMSO	6.7+/-2.2	56.2+/-18.5	Monomer
	Chlorobiocin	6.7+/-2.0	58.7+/-17.3	Monomer
	Coumermycin A1	6.7+/-1.9	56.7+/-15.8	Monomer
	Derrubone	7.8+/-3.0	82.3+/-31.2	Monomer-Dimer
	MDCC	6.2+/-1.0	46.8+/-7.9	Monomer
Hsp90αC	Buffer	9.1+/-3.0	117.7+/-38.2	Tetramer
	DMSO	12.9+/-4.2	265.6+/-86.4	Hexamer to Dodecamer
	Chlorobiocin	14.8+/-5.9	364.0+/-57.7	Dodecamer/Oligomer
	Coumermycin A1	17.4+/-5.5	533.5+/-383.7	Oligomer
	Derrubone	15.7+/-7.4	416.6+/-307.9	Oligomer
	MDCC	167.6+/-40.0	1.06E5+/-2.54E4	Oligomer/Polymerized
Hsp90α_FL	Buffer	15.7+/-3.3	415.8+/-89.1	Hexamer
	DMSO	20.3+/-9.4	757.4+/-499.5	Hexamer to Dodecamer
	Chlorobiocin	19.9+/-4.4	727.0+/-160.4	Hexamer to Dodecamer
	Coumermycin A1	18.3+/-5.0	595.3+/-162.8	Hexamer to Dodecamer
	Derrubone	19.0+/-5.7	651.4K+/-195.7K	Hexamer to Dodecamer
	MDCC	25.5+/-11.8	1300+/-599.6	Oligomer/Polymerized

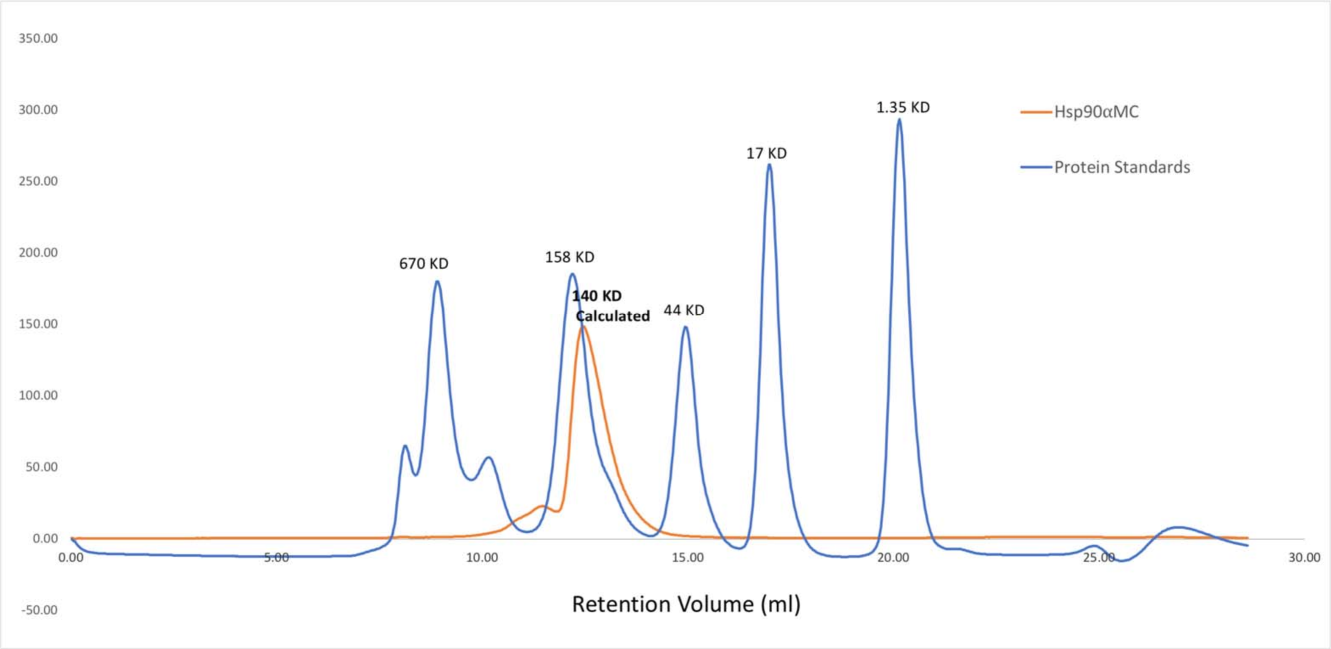

Figure S1 SEC chromatograph of Hsp90αMC protein. The calculated MW of Hsp90αMC is 140 kDa, representing a homodimer.

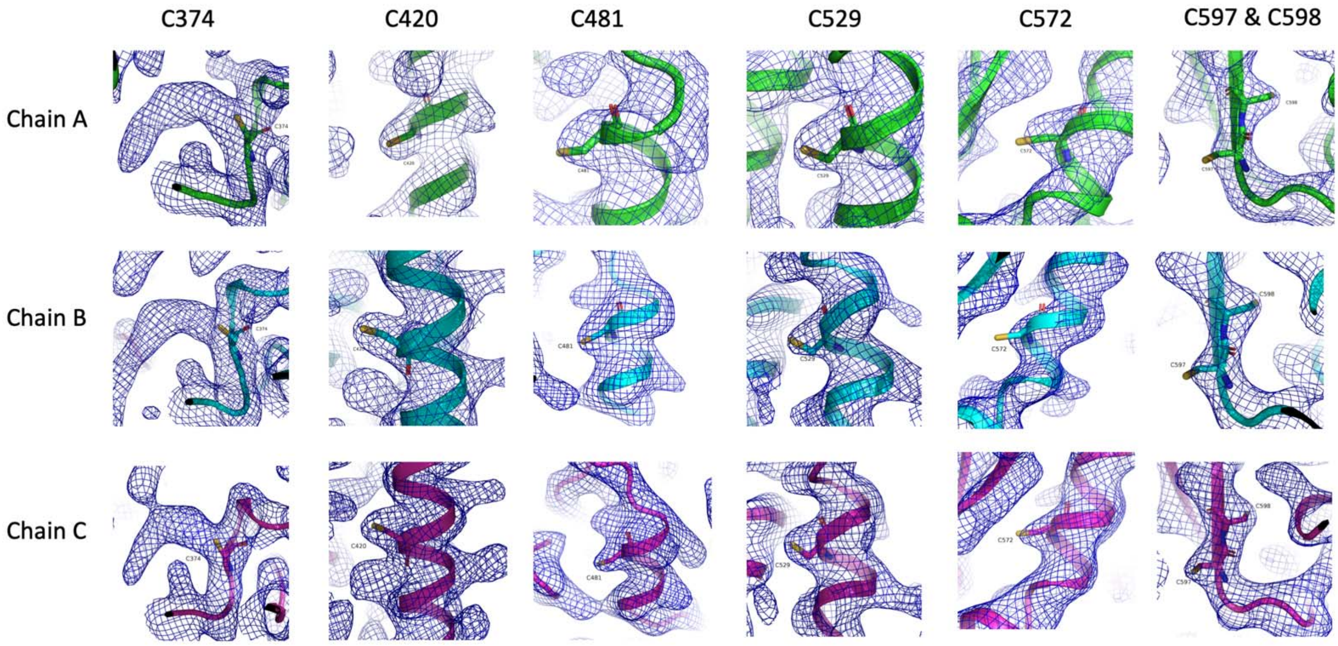

Figure S2 The local electron density maps (2mFo-DFc map, contour level of 1.0 σ) of all cystines in the crystal structure of chain A (green), chain B (cyan) and chain C (magenta).

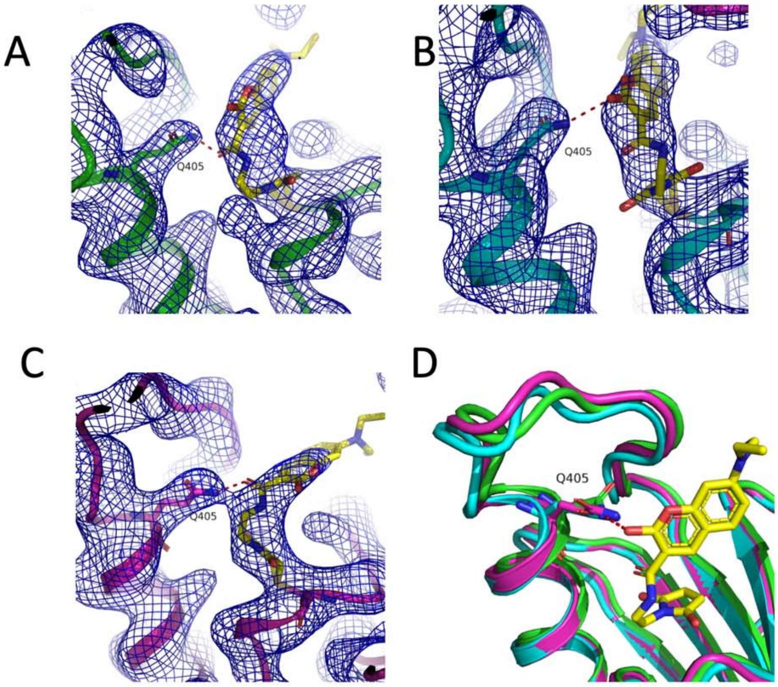

Figure S3 The local electron density maps (2mFo-DFc map, contour level of 1.0 σ) of residue Q405 of Hsp90α involved in H-bonding with MDCC in the crystal structure. A, chain A (green), B, chain B (cyan), C, chain C (magenta). (D) Structural alignment of Q405 in the three molecules.

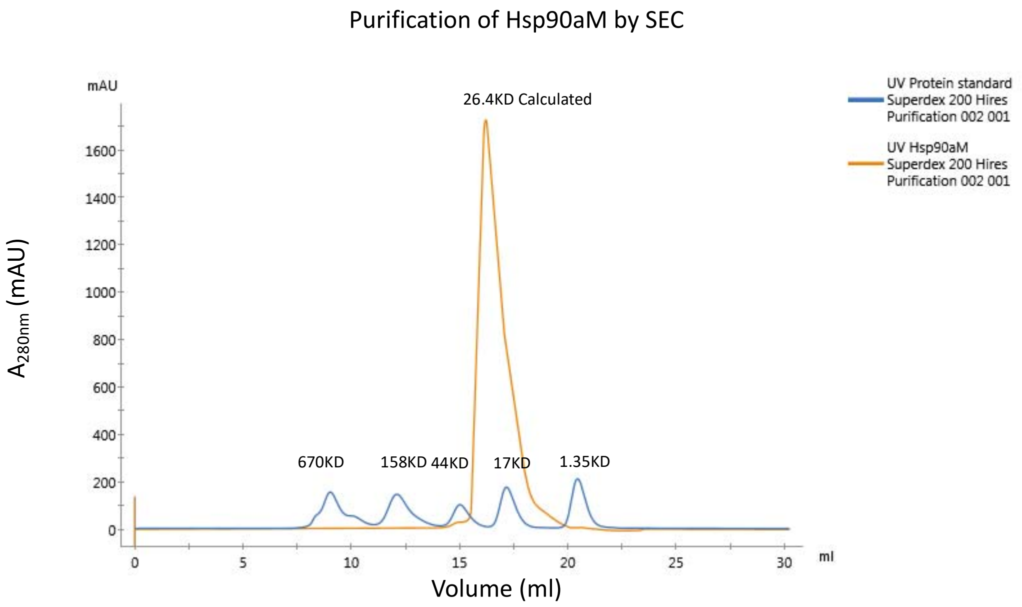

Figure S4 SEC chromatograph of Hsp90αM protein. The calculated MW of Hsp90αM is 26.4 kDa), representing a monomer.

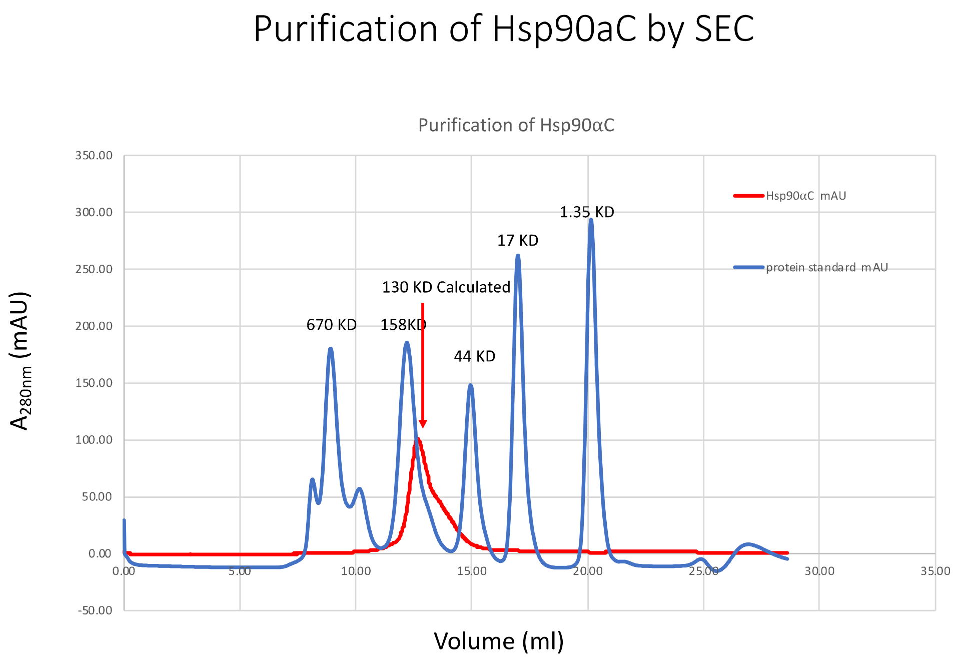

Figure S5 SEC chromatograph of Hsp90αC protein. The calculated MW of Hsp90αC is 130 kDa (theoretical MW: 31.0 kDa), representing a tetramer.

High-load Purification of Hsp90a_FL by SEC

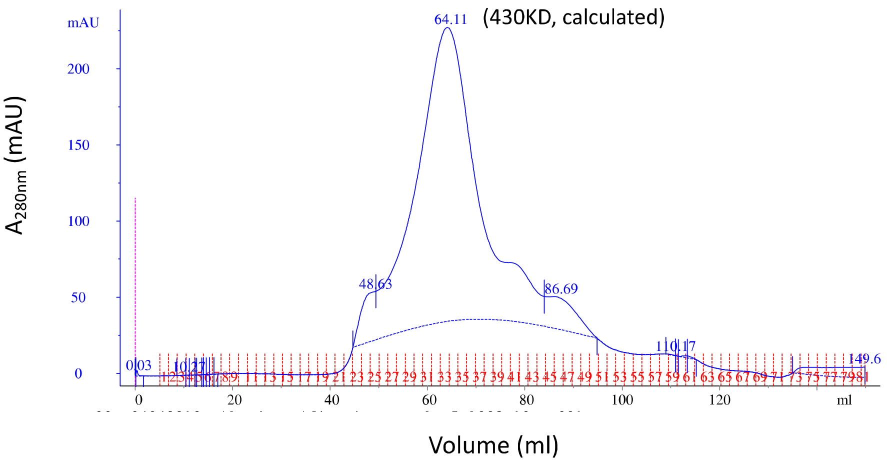

Figure S6 SEC chromatograph of Hsp90αFL protein on a prep grade superdex S200 column. The estimated MW is 430KD, representing a hexamer.

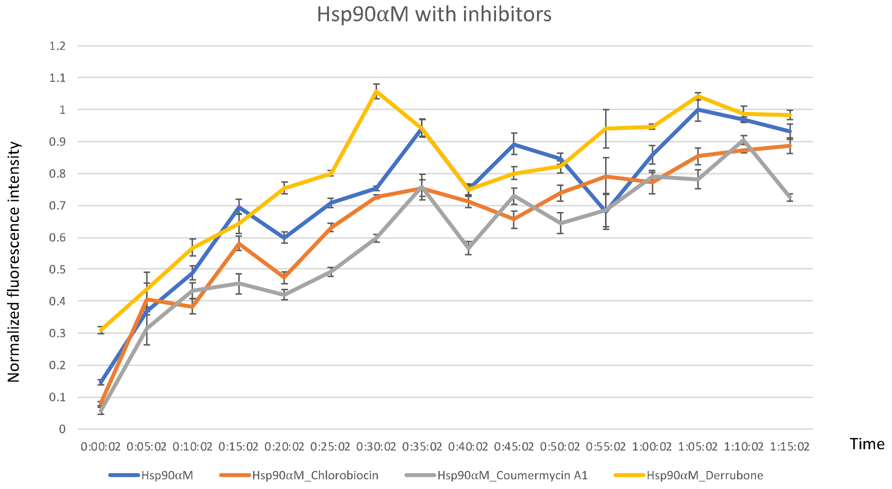

Figure S7 Fluorescence based competition assay. Various coumarin-core containing inhibitors were pre-incubated with Hsp90αM protein before adding MDCC, whose fluorescence intensity is monitored over time.

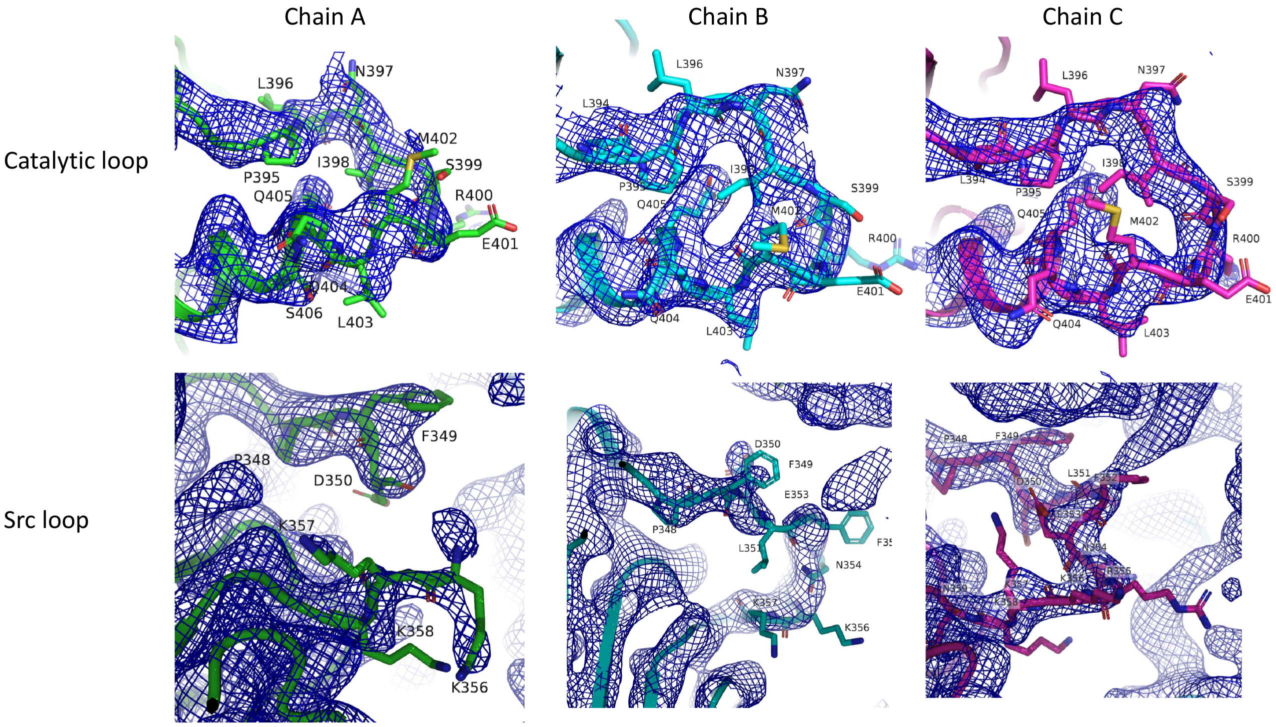

Figure S8 The local electron density maps (2mFo-DFc map, contour level of 1.0 σ) of the catalytic and src loops in the crystal structure of chain A (green), chain B (cyan) and chain C (magenta).

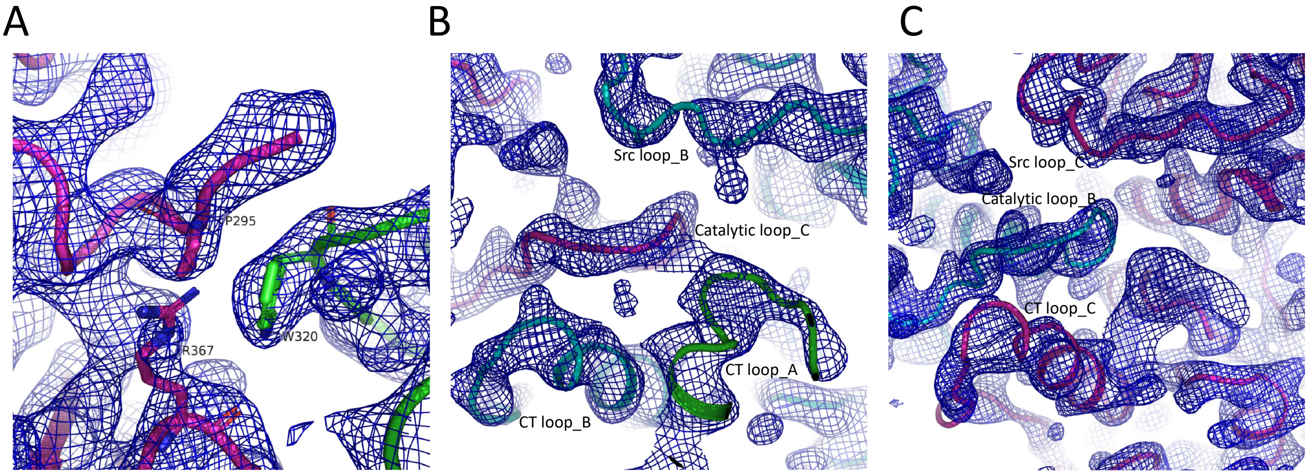

Figure S9 The local electron density maps (2mFo-DFc map, contour level of 1.0 σ) of hexameric interface, where molecule C interacts with molecules A and B. (chain A, green; chain B, cyan; chain C, magentas). (A) Hydrophobic association of P295 and R367 in molecule C with W320 in molecule A; (B) The catalytic loop in molecule C interacts with the Src and CT loops in molecule B, as well as the CT loop in molecule A; (C) The Src loop and CT loop in molecule C interact with the catalytic loop in molecule B.
